# Outcome assessment of intraoperative radiotherapy for brain metastases: results of a prospective observational study with comparative matched-pair analysis

**DOI:** 10.1007/s11060-023-04380-w

**Published:** 2023-07-21

**Authors:** Julian P. Layer, Motaz Hamed, Anna-Laura Potthoff, Cas S. Dejonckheere, Katharina Layer, Gustavo R. Sarria, Davide Scafa, David Koch, Mümtaz Köksal, Fabian Kugel, Molina Grimmer, Jasmin A. Holz, Thomas Zeyen, Lea L. Friker, Valeri Borger, F. Carsten Schmeel, Johannes Weller, Michael Hölzel, Niklas Schäfer, Stephan Garbe, Helmut Forstbauer, Frank A. Giordano, Ulrich Herrlinger, Hartmut Vatter, Matthias Schneider, L. Christopher Schmeel

**Affiliations:** 1 Department of Radiation Oncology, University Hospital Bonn, Venusberg-Campus 1, 53127 Bonn, Germany; 2 Institute of Experimental Oncology, University Hospital Bonn, Bonn, Germany; 3 Department of Neurosurgery, University Hospital Bonn, Bonn, Germany; 4 Division of Clinical Neuro-Oncology, Department of Neurology, University Hospital Bonn, Bonn, Germany; 5 Institute of Neuropathology, University Hospital Bonn, Bonn, Germany; 6 Department of Neuroradiology, University Hospital Bonn, Bonn, Germany; 7 Oncology Practice Network Troisdorf, Troisdorf, Germany; 8 Department of Radiation Oncology, University Medical Center Mannheim, Mannheim, Germany; 9 DKFZ-Hector Cancer Institute of the University Medical Center Mannheim, Mannheim, Germany; 10 Mannheim Institute of Intelligent Systems in Medicine (MIISM), Medical Faculty Mannheim, University of Heidelberg, Mannheim, Germany

**Keywords:** Surgery for brain metastases, Intraoperative radiotherapy, IORT, Local tumor control, Survival, Adjuvant radiotherapy

## Abstract

**Purpose:**

Intraoperative radiation therapy (IORT) is an emerging alternative to adjuvant stereotactic external beam radiation therapy (EBRT) following resection of brain metastases (BM). Advantages of IORT include an instant prevention of tumor regrowth, optimized dose-sparing of adjacent healthy brain tissue and immediate completion of BM treatment, allowing an earlier admission to subsequent systemic treatments. However, prospective outcome data are limited. We sought to assess long-term outcome of IORT in comparison to EBRT.

**Methods:**

A total of 35 consecutive patients, prospectively recruited within a study registry, who received IORT following BM resection at a single neuro-oncological center were evaluated for radiation necrosis (RN) incidence rates, local control rates (LCR), distant brain progression (DBP) and overall survival (OS) as long-term outcome parameters. The 1 year-estimated OS and survival rates were compared in a balanced comparative matched-pair analysis to those of our institutional database, encompassing 388 consecutive patients who underwent adjuvant EBRT after BM resection.

**Results:**

The median IORT dose was 30 Gy prescribed to the applicator surface. A 2.9% RN rate was observed. The estimated 1 year-LCR was 97.1% and the 1 year-DBP-free survival 73.5%. Median time to DBP was 6.4 (range 1.7–24) months in the subgroup of patients experiencing intracerebral progression. The median OS was 17.5 (0.5-not reached) months with a 1 year-survival rate of 61.3%, which did not not significantly differ from the comparative cohort (p = 0.55 and p = 0.82, respectively).

**Conclusion:**

IORT is a safe and effective fast-track approach following BM resection, with comparable long-term outcomes as adjuvant EBRT.

**Supplementary Information:**

The online version contains supplementary material available at 10.1007/s11060-023-04380-w.

## Introduction

Over the course of their disease, up to 40% of cancer patients develop brain metastases (BM) [1]. With novel therapeutic options prolonging their overall survival (OS) [2–5], the diagnostic incidence of BM and risk of local recurrence are increasing [6, 7]. Although BMs do nowadays not necessarily impact overall survival [8, 9], local treatment is critical to prevent or stabilize neurological deterioration and impairment of quality of life (QOL) [10, 11]. If feasible, large or symptomatic lesions require surgical intervention. Adjuvant radiation therapy (RT) of both potential tumor remnants and the resection cavity improves local control rates (LCR) [12–14]. Considering the tumor localization, histology and volumes, common RT regimens apply stereotactic external-beam RT (EBRT) of one (stereotactic radiosurgery, SRS) to seven fractions (fractionated stereotactic radiotherapy, FSRT) either before resection or afterwards, following adequate wound healing and recovery from surgery [14–18]. As an alternative, low-energy intraoperative RT (IORT) has increasingly gained attention in the past years. Initial reports suggest promising LCR [19, 20] and a favorable safety profile [21] with a comparatively lower radiation necrosis (RN) incidence [22]. Available data are solely based on retrospective single institution experiences, with a radiation oncology focus on dosage and technical aspects of the IORT approach [23, 24]. Nonetheless, its safety profile has been previously explored in brain tissue for treating glioblastoma [25, 26] and its efficacy is currently evaluated in a phase III trial (NCT02685605). Several advantages of IORT include a steep dose gradient, improved healthy brain tissue sparing [27] and avoiding RT target-volume delineation challenges caused by post-surgery tissue alterations. The instant application of local high dose RT to the tumor bed may prevent early repopulation of residual microscopic tumor. Furthermore, an accelerated completion of the interdisciplinary BM treatment eases a faster recovery, shorter in hospital-times and earlier initiation of subsequent systemic treatments.

We previously reported on a favorable perioperative safety profile of patients receiving IORT for BM in a matched-pair fashion with 388 BM patients who underwent conventional post-surgical RT [28]. Here, we report on their clinical long-term outcome and assess their survival in comparison to the same matched institutional cohort.

## Methods

### Patients

The study collected data from consecutively recruited patients admitted to the Neurosurgical Department of the University Hospital Bonn between November 2020 and October 2021, who had undergone surgical resection of BM combined with IORT. In all cases, BM were histopathologically confirmed. At a weekly tumor board meeting, interdisciplinary consensus was used to determine the treatment strategies for each patient individually [29]. Treatment plans were also coordinated with the referring physicians and considered the patient’s past oncological therapies. Besides receiving a histopathological diagnosis in case of cancer of unknown primary, criteria for surgical resection were presence or severe risk of acute neurological impairment or clinically significant mass effects as abnormal intracranial pressure or hemispheric shift. In case of multiple BMs, only the clinically manifest lesion was considered for surgical removal to prevent mass effects and tumor-related hydrocephalus [28, 30]. Clinical inclusion criteria for IORT were gross total resection, intraoperative confirmation of BM on frozen tumor sections, no previous intracerebral irradiation and fulfillment of dose constraints as described below. The data were prospectively collected and managed using SPSS (version 25, IBM Corp., Armonk, NY). Informed consent was obtained from all patients. The collected information included, among others, sociodemographic characteristics, primary tumor location, radiological and histopathological characteristics of the intracranial metastatic lesions, baseline functional status. The Karnofsky performance score (KPS) was used to classify the patients according to their functional status at admission. A stratification cut-off of 70 was chosen according to Péus et al. with regard to the patient’s ability to carry on their normal activity and work [31]. Diagnostic-Specific Graded Prognostic Assessment (DS-GPA) [32] scores were calculated by standard procedures. The study was conducted in accordance with the principles of the Declaration of Helsinki and approved by the Ethics Committee of the University Hospital Bonn (approval number: 018/21 and 057/22).

### IORT

Preoperative contrast-enhanced T1-weighted MRI imaging was used to provide 3D image guidance for both surgery and radiation treatments. Optic nerves, chiasm, and brain stem were identified preoperatively and intraoperatively as organs at risk (OARs) for IORT and delivered doses were defined based on dose-depth template profiles corresponding to each applicator diameter. The INTRABEAM^®^ 600 (Carl Zeiss Meditec AG, Oberkochen, Germany) was used to deliver IORT with a spherical applicator ranging from 1.5 to 5.0 cm diameter by application of nominal 50 kV photons at a standard dose of 30 Gy prescribed to the applicator surface. Decreasing the prescribed dose down to 16 Gy was acceptable in case of OAR doses exceeding the constraints of 12 Gy to the optical system or 12.5 Gy to the brain stem following the QUANTEC (Quantitative Analyses of Normal Tissue Effects in the Clinic) recommendations [33] considering the specific (1.3–1.5 times higher) RBE of low energy photons. In individual cases, an anatomical positioning of the applicator required consideration of further OAR that were not regularly assessed, e.g., cochlea or thalamus, with equal consideration of the QUANTEC recommendations.

### Follow-up

All patients had regular follow-up (FU) visits including physical examination and magnetic resonance imaging (MRI). MRI assessments were performed according to the RANO criteria by board-certified radiologists. In case of uncertain clinical or radiographic response, the interdisciplinary neuro-oncological tumor board was consulted and a combined decision was taken upon findings. The following conditions qualified for diagnosis of RN: (1) after initial suspected progressive disease (PD), a minimum of two FU MRI time points showed no sign of ongoing PD; (2) advanced MRI incorporating dynamic susceptibility contrast (DSC) or diffusion weighted imaging (DWI) was concordantly suggestive of RN; (3) RN was confirmed histopathologically after surgery.

### Study endpoints

The primary endpoints of the study were RN rates and cumulative 1 year-LCR. The secondary endpoints were DBP, 1 year-OS rates and estimated OS. Local control was defined as the absence of MRI-radiographic PD in or surrounding the previously irradiated BM resection cavity and calculated from the day of surgery until the date of PD. Patients lost to FU or deceased prior to radiographic progression were censored at the last FU time point. OS was defined as the time interval between the date of surgery and the date of either the last FU (censored) or death.

### Matching procedure

The study performed a propensity score matching, which involved matching a cohort of 35 patients who received IORT with a cohort of 388 patients who underwent surgery for BM followed by EBRT (patient characteristics provided in Suppl. Table 1). The matching was performed at a ratio of 1:2, and the statistical computing program R (version 4.1.2; The R Foundation for Statistical Computing, https://www.r-project.org/) was used for the analysis as previously described [28]. The group of EBRT patients included all patients aged 18 years or older who underwent surgery for BM at the University Hospital Bonn neuro-oncological center between 2013 and 2018, and who did not receive IORT but EBRT (SRS, FSRT or whole brain radiotherapy (WBRT)) during that period. The study aimed to increase the robustness of the data by selecting known prognostic parameters, such as age [34], KPS and Charlson comorbidity index (CCI) at admission [34–36], tumor entity, and the status of solitary versus multiple BM [35], for matching. The balance of these parameters was measured and visualized to ensure that the two groups were comparable. A jitter plot was used to display the distribution of propensity scores. The study protocol for retrospective data collection was approved by the local Ethics committee (approval number: 250/19 and 057/22).

### Statistics

The computer software packages used for the data analyses were SPSS and GraphPad Prism (version 9, GraphPad Software, Boston, MA). Fisher’s exact test was used to analyze categorical variables, which were presented in contingency tables. The Mann–Whitney U test was used to compare continuous variables, as the data were not normally distributed. Statistical significance was defined as a p-value of less than 0.05.

## Results

### Patient and tumor characteristics

Between November 2020 and October 2021, 35 consecutive BM patients receiving IORT to the resection cavity were enrolled. Their median age was 63 (range 43–80) years and the median KPS was 80 (50–100). Of note, 29% of patients had a KPS < 70. The median DS-GPA score was 2 (0–4). The most frequent BM localization was the frontal lobe (37.1%) followed by the occipital lobe (25.7%). Most histopathology results corresponded to non-small cell lung cancer (NSCLC, 54%), followed by melanoma (11%) and breast cancer (6%). With a range of 2 to 10 intracranial lesions, 15 patients (43%) suffered from multiple BM at the time of surgery. Further details on patient characteristics can be found in Table 1.

**Table 1:** Patient and tumor characteristics*

	n = 35
Median age (IQR) (in yrs)	63 (54–71)
Female sex	19 (54.3)
Primary site of cancer	
Lung	21 (60)
Melanoma	4 (11)
Kidney	4 (11)
Breast	2 (6)
Others	4 (11)
Multiple BMs	15 (43)
Preoperative KPS < = 70	14 (40)
Median DS-GPA score	2 (0–4)
Concomitant systemic treatment	3 (9)
Median dose of IORT (in Gy)	30 (16–30)
Median duration of IORT (in min)	18.2 (6.9–49)

*BM* brain metastasis, *CCI* Charlson comorbidity index, *DS-GPA* diagnosis-specific graded prognostic assessment, *Gy* gray, *IQR* interquartile range, *KPS* Karnofsky performance score, *mOS* median overall survival, *n* number of patients, *yrs* years

^*^Values represent the number of patients unless indicated otherwise (%)

### Treatment and dosimetry

No dose constraints were exceeded and all patients completed treatment. The median IORT duration was 18:12 (6:56–49:00) min and the median prescription dose was 30 (16–30) Gy. The median applicator size was 2.5 (1.5–5.0) cm. The brainstem and the optic tracts (optic nerves and chiasm) were regularly assessed as OARs. Doses to other structures were negligible and therefore not considered relevant for this report. The median distance from the applicator surface was 35.5 (10–65) mm to the brainstem and 60 (13–70) mm to the optic tracts, with a median estimated OAR dose exposure of 0.7 (0.0–6.0) Gy and 0.0 (0.0–4.4) Gy, respectively.

### Radiation necrosis rate, local tumor control and distant brain progression

**Fig. 1:**
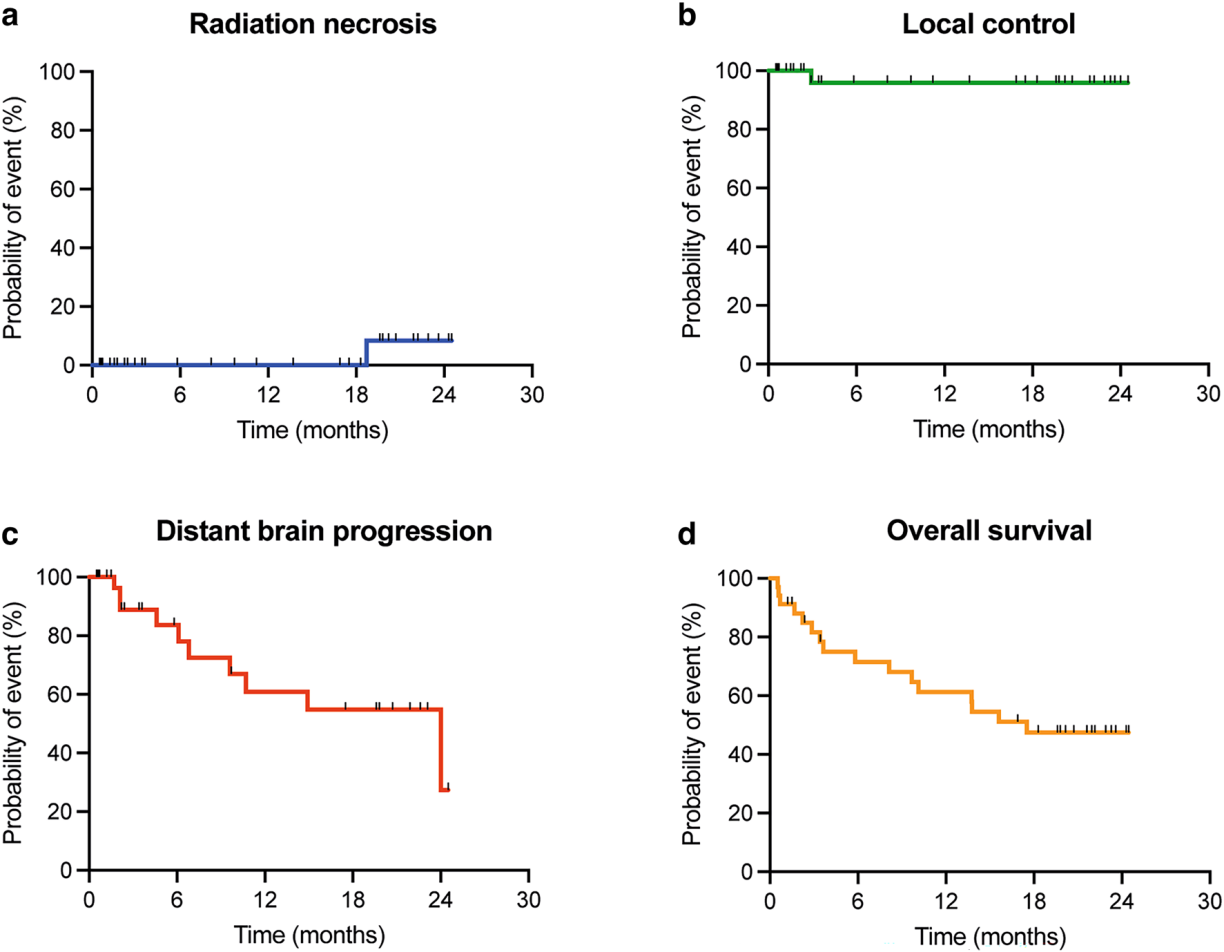
Outcome of IORT patients. Kaplan–Meier curves for **a** radiation necrosis, **b** local control, **c** distant brain progression and **d** overall survival

In all patients, a gross total resection was achieved. After a median FU of 10.4 (0.5–24.5) months and a median imaging FU of 7.9 (0.1–24.4) months incorporating a median of 6 (1–13) MRI assessments, only one RN event was noted at 18.7 months. Hence, an overall RN rate of 2.9% was observed (Fig. 1a). As this patient’s RN was a grade 2 event, only mild conservative management was initiated and subsequently led to clinical remission. Of note, the patient did not experience distant intracranial progression and is still alive and systemically stable after 23.2 months of FU.

A second patient showed local recurrence after 2.9 months, in addition to previous distant intracranial progression. The latter led to clinical deterioration and subsequent exitus. The overall IORT 1-year LCR was 97.1% (Fig. 1b). With an overall distant brain progression rate of 29.4%, the median DBP-free survival (DBPS) was 24 (0.5-not reached) months and the 1 year-DBPS 73.5% (Fig. 1c). The median time to DBP was 6.4 (range 1.7–24) months in the subgroup of patients experiencing distant intracranial progression. Leptomeningeal spread occurred in 5.7% of cases (2 cases), after 18.2 and 21.9 months, respectively.

### Survival and comparison to matched EBRT cohort

**Fig. 2:**
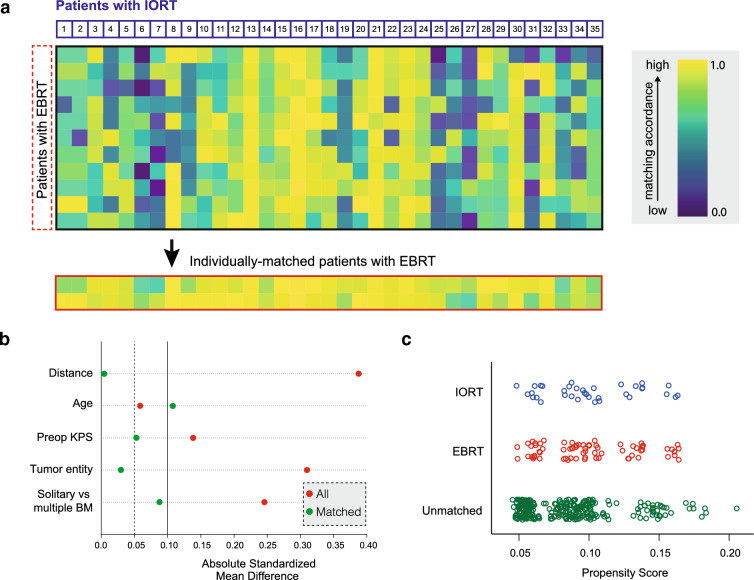
Graphical visualization of the applied matching procedure. **a** Comparative matched pair analysis at a ratio of 1:2 identifies 70 out of 388 patients with resected BM not receiving IORT who individually correspond to the present series of 35 patients with resected BM undergoing IORT. Heat map as color-coded illustration of the matching strategy of patients not receiving IORT to IORT cases stratified by age, KPS at admission, tumor entity and solitary versus multiple BM as matching parameters. The red box illustrates individually-matched patients without IORT. **b** Love plot demonstrating the balance of the matching analysis for each matching parameter determined by the standardized mean differences. **c** Illustration of propensity scores obtained as described in **a** for matched (blue: IORT; red: EBRT) and unmatched BM patients (green). *BM* brain metastasis, *EBRT* external beam radiation therapy; *IORT* intraoperative radiotherapy, *KPS* Karnofsky performance score.

**Fig. 3:**
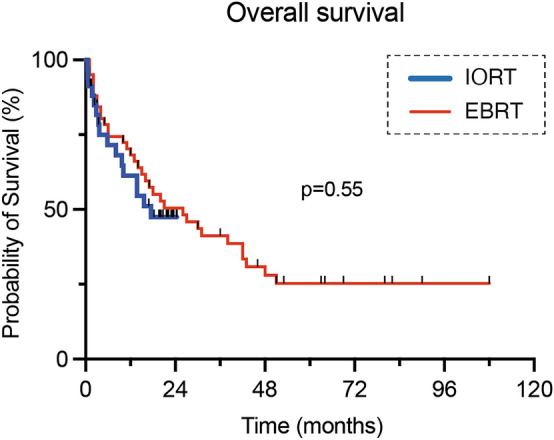
Kaplan Meier survival curves for patients with surgically-treated BM stratified for IORT versus EBRT. *EBRT* external beam radiation therapy, *IORT* intraoperative radiotherapy

For the IORT cohort, the median OS was 17.5 (0.5-not reached) months and the 1 year-survival rate 61.3% (Fig. 1d). 70 patients from the institutional database of patients, who underwent surgery with subsequent EBRT (Suppl. Table 1) and individually corresponded to the present series were matched at a ratio of 1:2 to those receiving IORT (Fig. 2). The two patient populations did not differ significantly by the matching variables age (p = 0.74), KPS (p = 0.88), primary site of cancer (p = 1.00) and frequency of multiple BM (p = 0.68). Concomitant systemic treatment was equally distributed (p = 0.99). With 61.3% versus 68.2%, the 1 year-survival was not significantly different between IORT and EBRT, respectively (p = 0.82; Table 2). Furthermore, the median OS was comparable with 17.5 months and 26 months, in each respective cohort (p = 0.55; Fig. 3).

**Table 2:** Comparative matched pair analysis on survival outcome in patients with surgically-treated BM stratified for IORT versus EBRT*

	Surgery with IORT n = 35	Surgery with EBRT n = 70	p-value
Matching variables			
Age (years)	63 (54–71)	63 (57–70)	0.74
Preoperative KPS	80 (60–90)	80 (70–90)	0.88
Primary site of cancer			1.0
Lung Cancer	21 (60)	41 (59)	
Others	14 (40)	29 (41)	
Solitary vs. multiple			0.68
Multiple	15 (43)	27 (39)	
Solitary	20 (57)	43 (61)	
Outcome parameters			
1 year-survival	19/35** (61.3)	33/70*** (68.2)	0.82
mOS (months)	17.5	26	0.55

*BM* brain metastasis, *CCI* Charlson comorbidity index, *EBRT* external beam radiation therapy, *Gy* gray, *IQR* interquartile range, *KPS* Karnofsky performance score, *mOS* median overall survival, *n* number of patients

^*^Values represent the number of patients unless indicated otherwise (%)

^**^2 of 35 patients censored with lost to follow-up < 12 months (33 patients with event at any time)

^***^17 of 70 patients censored with lost to follow-up < 12 months (53 patients with event at any time)

## Discussion

IORT following BM resection is an emerging alternative to adjuvant EBRT, but long-term experience and efficiency are yet to be established. Taken together with our previous study [28], this is the first report on IORT for BM that covers both short and long-term clinical FU of a consecutive patient cohort and matches and compares their survival outcomes to those of EBRT.

There is consensus on the beneficial effect of adjuvant RT on local control after BM resection [14, 16, 37]. However, depending on the individual clinical context, it remains controversial which RT sequencing and technique achieves best long-term outcomes at lowest toxicity levels. Despite providing convincing BDFS [38, 39], WBRT was abandoned due to an inferior toxicity profile [15, 40–42] in comparison to modern stereotactic RT approaches. Accordingly, previous intracavitary treatment modalities, such as permanent intracerebral radio-isotopic seed implantation, yielded very good LCRs, [43–45] yet are prone to induce RN [46, 47]. Moreover, arterial occlusion [48], seed detachment and necessity of subsequent re-surgery could arise. For stereotactic RT, reports on LCR and toxicity differ significantly depending on entities, BM volume and number, but also the fractionation scheme [14, 15, 17, 18, 49]. Besides classical outcome parameters, patient-centered factors such as reduction of hospitalization times, timely treatment access and quality of life have become increasingly important both from patient-centered and socioeconomic view points. This applies in particular to BM patients in a palliative care setting that may suffer from neurological impairment along with a limited life expectancy. Additionally, most patients from our collective were first diagnosed with BM during staging of an extracranial primary tumor. For these patients, swiftness is particularly important, since at time of brain surgery they often still require completion of staging examinations and the initiation of systemic treatment [50]. IORT can expedite these urgent subsequent steps by approximately two to 3 weeks. Furthermore, patients at first diagnosis of metastatic cancer [51], especially with favorable prognostic factors like solitary BM [52], are likely to experience DBP requiring reirradiation to potentially closely located brain structures. The specific physical features of 50 kV IORT provide an increased linear energy transfer and a higher relative biological effectiveness [53] with steep dose gradients allowing both optimized tumoricidal effect and OAR sparing. Thus, patients may benefit from preservation of neurological functions and improved subsequent reirradiation options. The main disadvantage of IORT is a lack of dose modulation options that render certain anatomic conditions challenging. Therefore, it is not surprising that, in line with previous reports [20], most of the BM treated in this cohort were located either craniofrontal or occipital.

Consistent with our previously reported perioperative safety profile [28], we here report a favorable overall RN rate of just 2.9% after IORT. This is an improvement in comparison to adjuvant SRS or FSRT where RN rates typically range between 8% [14] to more than 20% [49], but also to some previous series of IORT patients. While Kahl et al. reported 2.5% [22], Cifarelli et al. noted a RN rate of 7% [19] and Diehl et al. of 11.1% [20]. Of note, the latter also included few patients receiving additional post-surgery stereotactic radiosurgery. In line with previous reports, we found only a comparably low incidence of leptomeningeal spread after IORT [19, 22]. This may be an additional clinical advantage of IORT over other RT techniques that requires further scientific attention. Our observed 1-year LCR of 97.1% compares well with the 94% observed by Cifarelli et al. [19] and outperforms most studies on both adjuvant and definitive SRS or FSRT with rates roughly between 80 to 90% [14, 17, 18, 49, 54–56]. Definitive SRS of BM is the primary alternative option to resection when systemic treatment delays are to be avoided. Both effectiveness and safety of single fraction EBRT mainly depend on lesion volume [55]. Compared to SRS only [56], our data indicate a superior LCR and RN rate for IORT of BM > 2 cm, while equally avoiding additional treatment times following surgery.

By matched pair analysis, we demonstrated comparable long-term survival outcomes of EBRT and IORT. The 1 year-survival rate of 57% reported here is also within the range of previous reports for IORT [20, 22]. Meanwhile, despite being marginally different, the matched cohort exhibited outstanding long-term survival. Many of the patients from this cohort surpassed a FU that timewise cannot be achieved yet for the IORT group and, thus, long-term survivors are censored at an earlier time point in the latter. In addition, there are remaining risk factors that could not be adjusted between the groups. While age, CCI, KPS and singularity of BM were considered as matching factors, DS-GPA scores were not. Depending on the tumor entity, this score covers further disease-specific risk factors. However, DS-GPA scores do not qualify for matching analyses as they are not applicable to all tumor entities, nor are they prognostically comparable between different entities [32]. The IORT cohort had a relatively low median DS-GPA score of only 2 and included a total of 25.7% of patients with at least 3 BM. Regardless of these unfavorable prognostic factors, the IORT cohort achieved outstanding local control as well as convincing RN rates in comparison to previous reports, while demonstrating equal long-term outcomes compared to matched EBRT cases.

### Limitations

Although the present study had a prospective observational design, its interpretation should take into account several limitations. The most significant limitation is the relatively small sample size of 35 patients, which may impact the generalizability of the findings. Of note, IORT remains a novel treatment option for BM with only very limited data available from comparably sized patient collectives. As an additional methodological measure, using a matched-pair approach could have helped to mitigate some confounding factors when comparing the long-term outcome of patients undergoing EBRT and IORT to BM. However, certain confounding factors, such as different prognostic profiles according to each histology or variable systemic treatment effects, were not regarded. Moreover, since FU MRIs were frequently carried out in local centers using minimized imaging protocols lacking DSC and/or DWI, no reliable data on local control and RN rates were available for the comparative cohort, hence it could not be included in the analysis. Despite these limitations in sample size, the present study may suffice to conceive further large-scale, cross-regional databases to accurately evaluate the safety, feasibility, and efficacy of IORT in the setting of BM surgery. This is the most comprehensive investigation on an IORT patient cohort thus far, incorporating dosimetric aspects, perioperative mortality and RN rate, as well as survival and local control outcomes.

## Conclusions

IORT is a timely feasible fast-track approach for complementing surgical BM treatment, with long-term safety and control outcomes comparable to those of adjuvant stereotactic RT. On-going phase II and III studies will soon elucidate the actual role of IORT in this setting.

## Supplementary Information

Below is the link to the electronic supplementary material.

Supplementary file1 (DOCX 14 KB)

## Data Availability

The data presented in this study are available in this article. Further datasets generated during and/or analysed during the current study are available from the corresponding author on reasonable request.

## References

[1] Cagney DN, Martin AM, Catalano PJ (2017). Incidence and prognosis of patients with brain metastases at diagnosis of systemic malignancy: a population-based study. Neuro Oncol.

[2] Antonia SJ, Villegas A, Daniel D (2018). Overall survival with durvalumab after chemoradiotherapy in stage III NSCLC. N Engl J Med.

[3] Motzer RJ, Tannir NM, McDermott DF (2018). Nivolumab plus Ipilimumab versus Sunitinib in advanced renal-cell carcinoma. N Engl J Med.

[4] Larkin J, Chiarion-Sileni V, Gonzalez R (2019). Five-year survival with combined nivolumab and ipilimumab in advanced melanoma. N Engl J Med.

[5] Modi S, Jacot W, Yamashita T (2022). Trastuzumab deruxtecan in previously treated HER2-low advanced breast cancer. N Engl J Med.

[6] Nayak L, Lee EQ, Wen PY (2012). Epidemiology of brain metastases. Curr Oncol Rep.

[7] Davis FG, Dolecek TA, McCarthy BJ, Villano JL (2012). Toward determining the lifetime occurrence of metastatic brain tumors estimated from 2007 United States cancer incidence data. Neuro Oncol.

[8] Yamamoto M, Sato Y, Serizawa T (2012). Subclassification of recursive partitioning analysis class ii patients with brain metastases treated radiosurgically. Int J Radiation Oncol Biol Phys.

[9] Nieder C, Stanisavljevic L, Aanes SG (2022). 30-day mortality in patients treated for brain metastases: extracranial causes dominate. Radiat Oncol.

[10] Schödel P, Schebesch K-M, Brawanski A, Proescholdt M (2013). Surgical resection of brain metastases—impact on neurological outcome. IJMS.

[11] Verhaak E, Gehring K, Hanssens PEJ, Sitskoorn MM (2019). Health-related quality of life of patients with brain metastases selected for stereotactic radiosurgery. J Neurooncol.

[12] Kocher M, Soffietti R, Abacioglu U (2011). Adjuvant whole-brain radiotherapy versus observation after radiosurgery or surgical resection of one to three cerebral metastases: results of the EORTC 22952–26001 study. JCO.

[13] Lehrer EJ, Peterson JL, Zaorsky NG (2019). Single versus multifraction stereotactic radiosurgery for large brain metastases: an international meta-analysis of 24 trials. Int J Radiation Oncol Biol Phys.

[14] Eitz KA, Lo SS, Soliman H (2020). Multi-institutional analysis of prognostic factors and outcomes after hypofractionated stereotactic radiotherapy to the resection cavity in patients with brain metastases. JAMA Oncol.

[15] Brown PD, Ballman KV, Cerhan JH (2017). Postoperative stereotactic radiosurgery compared with whole brain radiotherapy for resected metastatic brain disease (NCCTG N107C/CEC·3): a multicentre, randomised, controlled, phase 3 trial. Lancet Oncol.

[16] Mahajan A, Ahmed S, McAleer MF (2017). Post-operative stereotactic radiosurgery versus observation for completely resected brain metastases: a single-centre, randomised, controlled, phase 3 trial. Lancet Oncol.

[17] Jhaveri J, Chowdhary M, Zhang X (2019). Does size matter? Investigating the optimal planning target volume margin for postoperative stereotactic radiosurgery to resected brain metastases. J Neurosurg.

[18] Layer JP, Layer K, Sarria GR (2023). Five-Fraction stereotactic radiotherapy for brain metastases—a retrospective analysis. Curr Oncol.

[19] Cifarelli CP, Brehmer S, Vargo JA (2019). Intraoperative radiotherapy (IORT) for surgically resected brain metastases: outcome analysis of an international cooperative study. J Neurooncol.

[20] Diehl CD, Pigorsch SU, Gempt J (2022). Low-energy X-Ray Intraoperative radiation therapy (Lex-IORT) for resected brain metastases: a single-institution experience. Cancers.

[21] Krauss P, Steininger K, Motov S (2022). Resection of supratentorial brain metastases with intraoperative radiotherapy Is it safe? Analysis and experiences of a single center cohort. Front Surg.

[22] Kahl K-H, Balagiannis N, Höck M (2021). Intraoperative radiotherapy with low-energy x-rays after neurosurgical resection of brain metastases-an Augsburg University Medical center experience. Strahlenther Onkol.

[23] Sarria GR, Smalec Z, Muedder T (2021). Dosimetric comparison of upfront boosting with stereotactic radiosurgery versus intraoperative radiotherapy for glioblastoma. Front Oncol.

[24] Dahshan BA, Weir JS, Bice RP (2021). Dose homogeneity analysis of adjuvant radiation treatment in surgically resected brain metastases: comparison of IORT, SRS, and IMRT indices. Brachytherapy.

[25] Giordano FA, Brehmer S, Mürle B (2019). Intraoperative radiotherapy in newly diagnosed glioblastoma (INTRAGO): an open-label, dose-escalation phase I/II trial. Neurosurgery.

[26] Sarria GR, Sperk E, Han X (2020). Intraoperative radiotherapy for glioblastoma: an international pooled analysis. Radiother Oncol.

[27] Herskind C, Ma L, Liu Q (2017). Biology of high single doses of IORT: RBE, 5 R’s, and other biological aspects. Radiat Oncol.

[28] Hamed M, Potthoff A-L, Layer JP (2022). Benchmarking safety indicators of surgical treatment of brain metastases combined with intraoperative radiotherapy: results of prospective observational study with comparative matched-pair analysis. Cancers.

[29] Schäfer N, Bumes E, Eberle F (2021). Implementation, relevance, and virtual adaptation of neuro-oncological tumor boards during the COVID-19 pandemic: a nationwide provider survey. J Neurooncol.

[30] Hamed M, Potthoff A-L, Heimann M (2023). Survival in patients with surgically treated brain metastases: does infratentorial location matter?. Neurosurg Rev.

[31] Péus D, Newcomb N, Hofer S (2013). Appraisal of the karnofsky performance status and proposal of a simple algorithmic system for its evaluation. BMC Med Inform Decis Mak.

[32] Sperduto PW, Kased N, Roberge D (2012). Summary report on the graded prognostic assessment: an accurate and facile diagnosis-specific tool to estimate survival for patients with brain metastases. JCO.

[33] Marks LB, Yorke ED, Jackson A (2010). Use of normal tissue complication probability models in the clinic. Int J Radiation Oncol Biol Phys.

[34] Heimann M, Schäfer N, Bode C (2021). Outcome of elderly patients with surgically treated brain metastases. Front Oncol.

[35] Schneider M, Heimann M, Schaub C (2020). Comorbidity burden and presence of multiple intracranial lesions are associated with adverse events after surgical treatment of patients with brain metastases. Cancers.

[36] Ilic I, Potthoff A-L, Borger V (2022). Bone Mineral density as an individual prognostic biomarker in patients with surgically-treated brain metastasis from lung cancer (NSCLC). Cancers.

[37] Patchell RA, Tibbs PA, Regine WF (1998). Postoperative radiotherapy in the treatment of single metastases to the brain: a randomized trial. JAMA.

[38] Sahgal A, Aoyama H, Kocher M (2015). Phase 3 trials of stereotactic radiosurgery with or without whole-brain radiation therapy for 1 to 4 brain metastases: individual patient data meta-analysis. Int J Radiation Oncol Biol Phys.

[39] Brown PD, Jaeckle K, Ballman KV (2016). Effect of radiosurgery alone vs radiosurgery with whole brain radiation therapy on cognitive function in patients with 1 to 3 brain metastases: a randomized clinical trial. JAMA.

[40] Soffietti R, Kocher M, Abacioglu UM (2013). A European organisation for research and treatment of cancer phase III trial of adjuvant whole-brain radiotherapy versus observation in patients with one to three brain metastases from solid tumors after surgical resection or radiosurgery: quality-of-life results. J Clin Oncol.

[41] Mulvenna P, Nankivell M, Barton R (2016). Dexamethasone and supportive care with or without whole brain radiotherapy in treating patients with non-small cell lung cancer with brain metastases unsuitable for resection or stereotactic radiotherapy (QUARTZ): results from a phase 3, non-inferiority, randomised trial. Lancet.

[42] Hong AM, Fogarty GB, Dolven-Jacobsen K (2019). Adjuvant whole-brain radiation therapy compared with observation after local treatment of melanoma brain metastases: a multicenter, randomized phase III trial. J Clin Oncol.

[43] Wernicke AG, Hirschfeld CB, Smith AW (2017). Clinical outcomes of large brain metastases treated with neurosurgical resection and intraoperative cesium-131 brachytherapy: results of a prospective trial. Int J Radiat Oncol Biol Phys.

[44] Raleigh DR, Seymour ZA, Tomlin B (2017). Resection and brain brachytherapy with permanent iodine-125 sources for brain metastasis. J Neurosurg.

[45] Yang L, Wang C, Zhang W (2022). Iodine-125 brachytherapy treatment for newly diagnosed brain metastasis in non-small cell lung cancer: a biocentric analysis. Front Oncol.

[46] Wowra B, Schmitt HP, Sturm V (1989). Incidence of late radiation necrosis with transient mass effect after interstitial low dose rate radiotherapy for cerebral gliomas. Acta Neurochir (Wien).

[47] Huang K, Sneed PK, Kunwar S (2009). Surgical resection and permanent iodine-125 brachytherapy for brain metastases. J Neurooncol.

[48] Bernstein M, Lumley M, Davidson G (1993). Intracranial arterial occlusion associated with high-activity iodine-125 brachytherapy for glioblastoma. J Neuro-Oncol.

[49] Doré M, Martin S, Delpon G (2017). Stereotactic radiotherapy following surgery for brain metastasis: predictive factors for local control and radionecrosis. Cancer/Radiothérapie.

[50] Potthoff A-L, Heimann M, Lehmann F (2023). Survival after resection of brain metastasis: impact of synchronous versus metachronous metastatic disease. J Neurooncol.

[51] Lutterbach J, Cyron D, Henne K, Ostertag CB (2008). Radiosurgery followed by planned observation in patients with one to three brain metastases. Neurosurgery.

[52] Sawrie SM, Guthrie BL, Spencer SA (2008). Predictors of distant brain recurrence for patients with newly diagnosed brain metastases treated with stereotactic radiosurgery alone. Int J Radiat Oncol Biol Phys.

[53] Liu Q, Schneider F, Ma L (2013). Relative biologic effectiveness (RBE) of 50 kV X-rays measured in a phantom for intraoperative tumor-bed irradiation. Int J Radiat Oncol Biol Phys.

[54] Lehrer EJ, Ahluwalia MS, Gurewitz J (2022). Imaging-defined necrosis after treatment with single-fraction stereotactic radiosurgery and immune checkpoint inhibitors and its potential association with improved outcomes in patients with brain metastases: an international multicenter study of 697 patients. J Neurosurg.

[55] Minniti G, Clarke E, Lanzetta G (2011). Stereotactic radiosurgery for brain metastases: analysis of outcome and risk of brain radionecrosis. Radiat Oncol.

[56] Minniti G, Scaringi C, Paolini S (2016). Single-fraction versus multifraction (3 × 9 Gy) stereotactic radiosurgery for large (>2 cm) brain metastases: a comparative analysis of local control and risk of radiation-induced brain necrosis. Int J Radiat Oncol Biol Phys.

