# Supplementary material for: Outcome assessment of intraoperative radiotherapy for brain metastases: results of a prospective observational study with comparative matched-pair analysis
Source: J Neurooncol. 2023 Jul 21;164(1):107–16. doi: 10.1007/s11060-023-04380-w (PMC10462513; doi:10.1007/s11060-023-04380-w)
Supplement: Supplementary file 1 — Supplementary file1 (DOCX 14 KB) [file 11060_2023_4380_MOESM1_ESM.docx]

**Suppl. Table 1.** Characteristics of the matched EBRT patients.

|  | n = 70 |
| --- | --- |
| Median age (IQR) (in years) | 63 (57-70) |
| Female sex | 38 (54) |
| Primary site of cancer |  |
| Lung | 41 (59) |
| Others | 29 (41) |
| Multiple BMs | 27 (39) |
| Preoperative KPS >= 70 | 53 (76) |
| Concomitant systemic treatment | 7 (10) |
| EBRT |  |
| Median BED_10Gy_ (range) (in Gy) | 53 (30-64) |
| FSRT | 40 (57) |
| WBRT | 19 (27) |
| SRS | 4 (6) |
| Normofractionated | 3 (4) |
| Deceased before completion | 4 (6) |

BED: biologically effective dose; BM: brain metastases; IQR: interquartile range; FSRT: fractionated stereotactic radiotherapy; KPS: Karnofsky performance score; SRS: stereotactic radiosurgery; WBRT: whole brain radiotherapy.
